# From static guidelines to dynamic personalization: a novel AI-driven evidence-based paradigm for low-concentration atropine prescribing in myopia control

**DOI:** 10.3389/fmed.2026.1912484

**Published:** 2026-09-07

**Authors:** Yu Liu, Qingqing Zhou, Ling Ma, Yan Chen, Liping Sun, Jili Hu, Hongxing Kan

**Affiliations:** 1 Scool of Medical Information Engineering, Anhui University of Chinese Medicine, Hefei, Anhui, China; 2 Department of Ophthalmology, Anhui Provincial Children’s Hospital, Hefei, Anhui, China; 3 Department of Ophthalmology, the First Affiliated Hospital of USTC, Division of Life Sciences and Medicine, University of Science and Technology of China, Hefei, Anhui, China; 4 Center for Xin’an Medicine and Modernization of Traditional Chinese Medicine of IHM, Anhui University of Chinese Medicine, Hefei, Anhui, China

**Keywords:** atropine, BLEU, chain-of-thought, GraphRAG, myopia, RAGAS, ROUGE

## Abstract

**Background:**

The rapid increase in the global incidence of myopia poses challenges for prevention and management. Evidence-based low-concentration atropine eye drops can effectively slow down the progression of myopia. However the massive and constantly updated research data place a burden on clinicians, especially in primary care or regions with limited resources, hindering knowledge integration and evidence-based decision-making. There is still a gap in patients' access to reliable and personalized guidance. To address this issue, we developed MyoATP-AI-powered evidence based decision support system specifically designed for low-concentration atropine myopia control, built upon a GraphRAG and chain-of-thought reasoning architecture.

**Methods:**

We systematically searched PubMed (2000–2025) for literature on “atropine, myopia”, screening 104 relevant clinical articles and constructing a specialized dataset. Based on this, we developed MyoATP-AI, an AI-assisted diagnostic system combining GraphRAG and chain-of-thought reasoning. The knowledge graph structure was optimized through community detection and graph embedding. System performance was evaluated using the BLEU, ROUGE, and RAGAS frameworks. Five ophthalmologists rated the system's safety, professionalism, fluency, comprehensiveness, and satisfaction. Finally, we compared the 2024 consensus opinion with MyoATP-AI.

**Results:**

MyoATP-AI demonstrated excellent performance in fidelity, relevance, and context recall. The automated scoring was high: BLEU (0.9479), ROUGE-1 (0.969), ROUGE-L (0.9677), fidelity (0.947), answer relevance (0.9587), and context recall rate (0.925). Experts gave highly positive evaluations of the system across all dimensions, indicating its great potential in clinical decision support and patient education, as it can provide safe, professional, fluent, and comprehensive responses.

**Discussion:**

MyoATP-AI is a knowledge graph-based question-answering system that integrates GraphRAG and CoT technology for low-concentration atropine control of myopia. It can provide accurate and useful clinical advice, which, on the one hand, can assist doctors in making evidence-based decisions, and on the other hand, can offer reliable and easily understandable medical guidance to patients. Future work will expand the dataset to enhance the system's applicability in different scenarios and help with the prevention and control of myopia.

## 1 Introduction

Myopia has become a major global public health issue. Predictions indicate that by 2050, nearly half of the global population (approximately 4.758 billion people) will suffer from myopia, with the number of severe myopia patients exceeding 1 billion (1). In China, the overall myopia rate among children and adolescents is as high as 51.9%, meaning that at least one out of every two teenagers is affected by myopia. This situation not only poses a threat to individual health but also poses a risk to the future development of the country (2). Multiple center randomized controlled trials (RCTs) have demonstrated that low-concentration atropine eye drops are highly effective in controlling myopia (3). However, for patients, which atropine concentration is the most effective remains unresolved: which concentration of atropine has the best efficacy and the lowest side effects? Although the 0.01% concentration of atropine has the widest usage range and the lowest side effects, its long-term efficacy is generally average. Compared to the fixed use of 0.01% concentration, can a higher concentration (such as 0.025%, 0.05%) be used or can personalized treatment plans be implemented? The issue of the optimal concentration directly hinders the maximum utilization of the potential of atropine treatment (4).

Determining the optimal concentration requires continuously updated high-level evidence (5). However, many valuable basic research studies often fail to be promptly and systematically translated into clinical “best practices”, resulting in wasted resources and missed opportunities for patient benefit (6). This highlights a significant bottleneck in traditional evidence-based medicine (EBM) regarding the generation and application of evidence (7).

The central challenge lies in the efficiency limits of the traditional EBM evidence generation model (8). Meanwhile, the large language models (LLMs) in artificial intelligence have demonstrated remarkable capabilities in natural language understanding, generation, reasoning, and knowledge integration, providing revolutionary tools to overcome these limitations of EBM (9). This study developed MyoATP AI, an evidence-based conversational artificial intelligence system based on large language models (LLMs), specifically designed to control myopia with low-concentration atropine. MyoATP AI has two functions: on the one hand, it provides “ decision support based on expert opinions” for clinicians, especially in areas with limited resources, to enhance the accuracy of diagnosis and treatment; On the other hand, it conveys accurate and easily understandable knowledge about myopia prevention to patients, thereby improving treatment compliance and self-management ability. Therefore, MyoATP AI has significant clinical and social value.

To ensure the accuracy, interpretability, and reliability of the answers, the system integrates a graph retrieval-enhanced generation mechanism based on knowledge graph enhancement (GraphRAG) and combines chain-of-thought (CoT) reasoning technology.

The main innovation of our research does not lie in the GraphRAG or CoT technologies themselves, but rather in the first systematic application of this architecture to a specific clinical challenge—the low-dose atropine administration for children with myopia. Although GraphRAG and CoT have been explored in other medical fields, there has never been any literature reporting their combination for clinical decision support related to atropine. Our specific contributions include:

We constructed a knowledge graph based on GraphRAG, specifically designed for low-concentration atropine literature;

We developed a CoT-enhanced reasoning process for selecting atropine concentrations, managing side effects, and adjusting treatment plans;

A comprehensive multi-level evaluation framework, combining automated indicators, RAGAS assessment, expert clinical evaluation, and guidelines comparison for this clinical context.

A large number of large language models focused on the medical field have emerged, such as Med-PaLM (10) (clinical reasoning), Med-Flamingo (11) (evidence-based responses), DoctorGLM (12), and models centered on traditional Chinese medicine like MedGPT (13), and “Zhongjing” (14). Although they show potential in tasks such as symptom analysis and treatment recommendations, medical question-answering systems still face many challenges, including inconsistent terminology, limited reasoning ability, “knowledge hallucination”, and issues related to data privacy and credibility (15).

The Retrieval-Augmented Generation (RAG) (16) technology, which bases responses on an external, authoritative knowledge base, has been proposed to enhance quality and credibility and is particularly suitable for medical scenarios with high evidence requirements (17). For instance, LiVersa (liver disease) (18), and multimodal RAG systems combined with medical images (19). Graph-based enhancement techniques, such as GraphRAG and MedGraphRAG (20), have further optimized knowledge representation and reasoning capabilities by leveraging entity relationships and graph reasoning. Although building a high-quality medical knowledge graph is costly (21), successful integration has been achieved in specialized fields such as dentistry (22). However, most current RAG methods still rely on text retrieval rather than deeply modeling the structure of medical knowledge. Therefore, combining RAG with medical knowledge graphs (KGs) is the key path to building an efficient and trustworthy artificial intelligence medical assistant.

In the field of ophthalmology, generative artificial intelligence demonstrates great potential (for instance, using generative adversarial networks (GANs) for retinal imaging, and utilizing deep language models (DeepDR-LLM) for diabetic retinopathy screening) (23). However, general models like GPT-4V still struggle to handle specific ophthalmic diagnoses (such as vitreoretinal diseases), especially in responding to open-ended questions (24). Liu etl developed a model based on the Transformer architecture to predict the progression of equivalent spherical refractive error and axial length in children and adolescents over the next ten years (25). This model can accurately answer the question “How much will an individual's myopia progress?” However, a crucial and clinically urgent issue remains unsupported by AI tools: “For a specific child, which concentration of atropine should be chosen, and how should the treatment plan be adjusted over time?” Although randomized controlled trials have provided population-level evidence on the average efficacy of different concentrations of atropine, there is currently no AI tool available to convert these aggregated evidence into individualized, evidence-based, and easily accessible clinical recommendations. To our knowledge, no AI system has been specifically designed to integrate the rapidly growing literature on low-concentration atropine and provide an interactive question-and-answer platform to assist clinicians and patients in making individualized decisions related to atropine. This gap constitutes the direct motivation for this study.

## 2 Materials and methods

### 2.1 Data preprocessing

**Figure 1:**
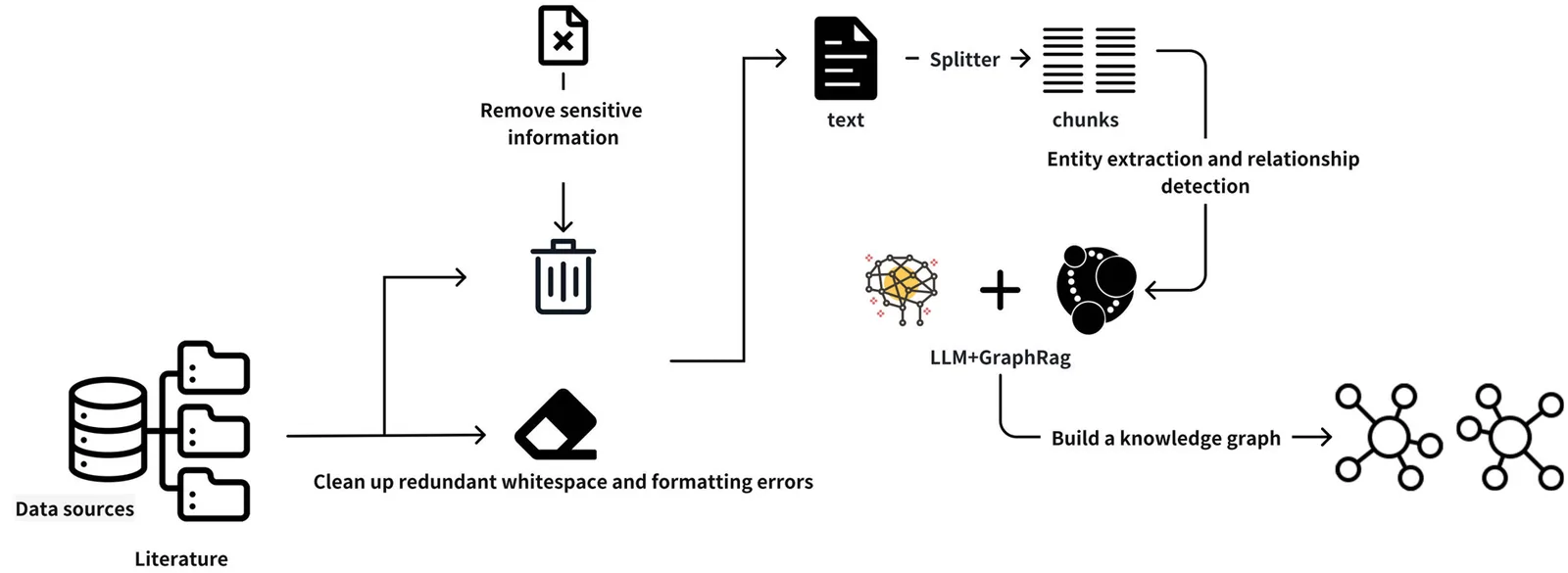
Data processing flowchart: data preprocessing removed sensitive information via regex, cleaned redundant blanks/format errors, and segmented text logically. The processed data was chunked for entity/relation extraction, constructing an atropine myopia treatment knowledge graph.

Using the keywords “atropine” and “myopia”, a comprehensive search was conducted in the PubMed database from 2000 to 2025 to ensure the inclusion of the latest and most comprehensive studies. Studies were included if they met all of the following criteria: (i) randomized controlled trials (RCTs); (ii) evaluated the efficacy and/or safety of atropine for myopia control; and (iii) reported at least one outcome measure related to myopia progression (e.g., spherical equivalent or axial length). Studies were excluded if they: (i) were not RCTs (e.g., case reports, case series, reviews, editorials, or observational studies without a control group); (ii) did not report pre- and post-intervention data on myopia progression; or (iii) had insufficient data for extraction. Two independent reviewers screened the titles and abstracts of all retrieved records. Full texts of potentially eligible articles were then assessed against the inclusion/exclusion criteria. Disagreements were resolved through discussion, with arbitration by a third reviewer when consensus could not be reached. This process ultimately yielded 104 eligible studies for knowledge graph construction. These articles almost cover all the clinical data on the effects of different concentrations of atropine on the myopia of teenagers, and take into account the influences of variables such as country, race, age and duration of medication. To meet the data requirements of the question-answering system based on GraphRAG technology, we first carried out strict preprocessing of the original documents. The specific workflow is shown in Figure 1.

### 2.2 Construction of MyoATP-AI

**Figure 2:**
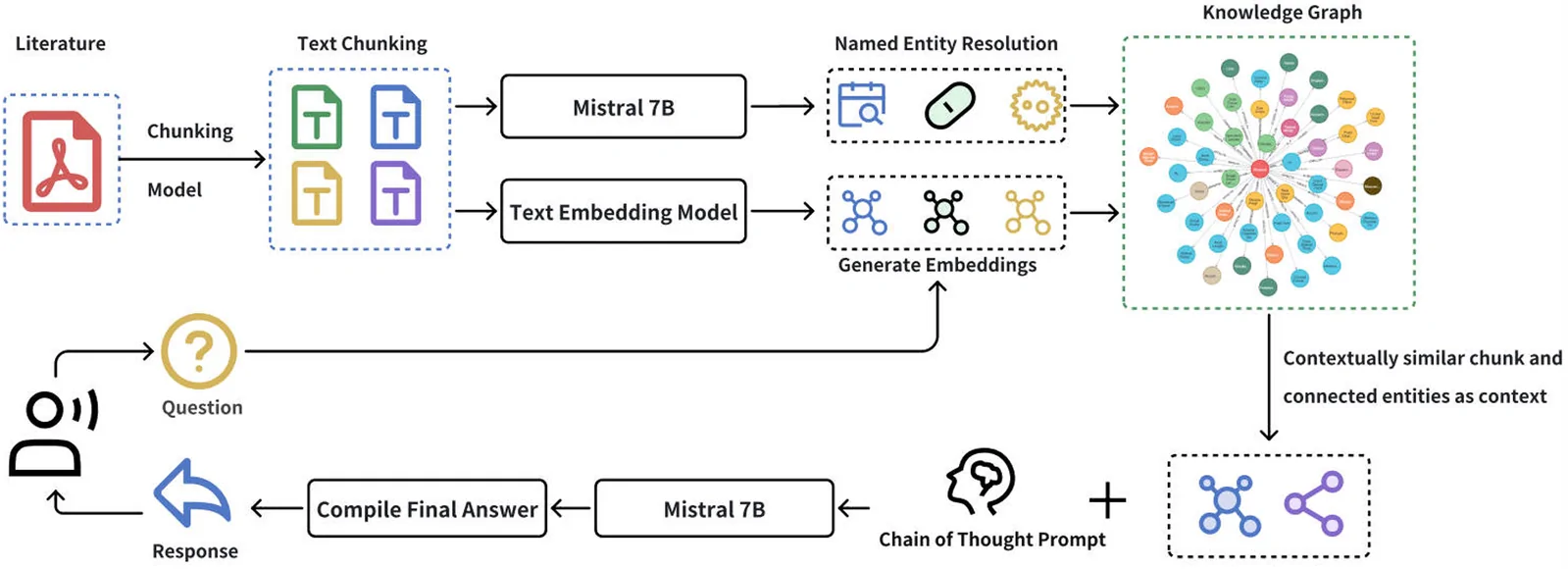
System implementation flowchart: the raw text is segmented and vectorized to extract entities and relationships for a knowledge graph. In the Q&A phase, the system retrieves relevant knowledge fragments from the graph, combines them with CoT prompts, and inputs them into LLMs to generate accurate answers.

MyoATP-AI is based on the open-source language model Mistral 7B and the GraphRAG framework. It innovatively incorporates the chain reasoning (CoT) strategy (26), which, through step-by-step logical reasoning, significantly enhances the analytical ability for the complex medical issue of “which low-concentration atropine can achieve the best effect in preventing and controlling myopia”. The implementation process of this system is shown in Figure 2.

#### 2.2.1 Mistral 7B model

The key innovation of this model lies in its architectural design: First, the group query attention (GQA) mechanism is used to significantly improves the inference speed and reduces GPU memory consumption by optimizing attention calculation. Secondly, it introduces the Sliding Window Attention (SWA), enabling the model to efficiently handle long sequence inputs with lower computational costs, thereby enhancing its ability to cope with complex contexts. However, in order to further improve the logical coherence and accuracy of the model when answering complex questions, especially in scenarios requiring multi-step reasoning, we plan to adopt the CoT prompt technology.

#### 2.2.2 Chain-of-thought (COT)

In MyoATP-AI, we have integrated two approaches: zero-sample CoT and a small sample CoT (27). The zero sample CoT prompts the model to conduct preliminary reasoning through prompts such as “Let's think step by step” before providing the final answer, thereby reducing direct guessing. To further enhance the model's professionalism and accuracy in the field of atropine, we adopted the small sample CoT method. This method guides the model to gradually analyze issues related to atropine, such as identifying the patient's condition, evaluating the atropine regimen (concentration/frequency), checking for side effects, and accordingly proposing comprehensive suggestions.

For consultation on treatment adjustments or discontinuation, the CoT prompts are designed to guide the model to follow a structured clinical reasoning path: The model will be sequentially required to evaluate the following: (i) the patient's baseline characteristics (age, baseline SE and AL); (ii) the duration of current treatment and the recent progression rate at the current atropine concentration; (iii) whether any adverse reactions have been reported; (iv) evidence from 104 articles' corpus regarding the rebound risk and post-discontinuation progression rate of relevant randomized controlled trials (RCTs); and (v) treatment options supported by evidence (continue treatment, reduce dosage or completely discontinue). This step-by-step reasoning ensures that the generated recommendations are clearly based on the retrieved evidence rather than relying on general heuristic methods.

By enabling the model to learn and imitate the expert-level reasoning process, MyoATP-AI is able to generate more profound and clinically logical responses, significantly improving the quality and reliability of the question-answering process.

### 2.3 Build MyoATP AI utilizing GraphRAG

#### 2.3.1 Construction of knowledge graph

In each text block, the Mistral 7B model is used to extract entities and relationships. After the extraction is completed, we optimize the knowledge graph by merging entities with the same name and type. At the same time, we integrate the relationships with the same source and target nodes into a unified description, thereby constructing a more concise and consistent representation of the knowledge graph. The specific implementation process can be seen in Supplementary Figure S1.

The extracted entities and relationships were subsequently stored in the graph database to complete the construction of the knowledge graph. The visualization results of Neo4j are shown in Supplementary Figure S2.

#### 2.3.2 Community analysis and summarization of knowledge graph

After constructing the knowledge graph, the Leiden community detection algorithm was applied for hierarchical segmentation to identify different granularity of entity communities. These communities represent different themes and sub-domains within the field of using low-concentration atropine for the prevention and control of myopia. After obtaining the community structure and graph embedding, we used a large language model to generate summary reports for each community (Supplementary Figure S3).

#### 2.3.3 Document vectorization and semantic relationship

To ensure document-level semantic analysis, we are associate each document with its corresponding text block and generate vector representations through document slicing embedding. By calculating the embeddings of the text blocks and taking their average, we can construct the vector representation of the document. This method helps to better understand the implicit relationships between documents in the subsequent question answering process (Supplementary Figure S4).

#### 2.3.4 Implement query and question—answering

**Figure 3:**
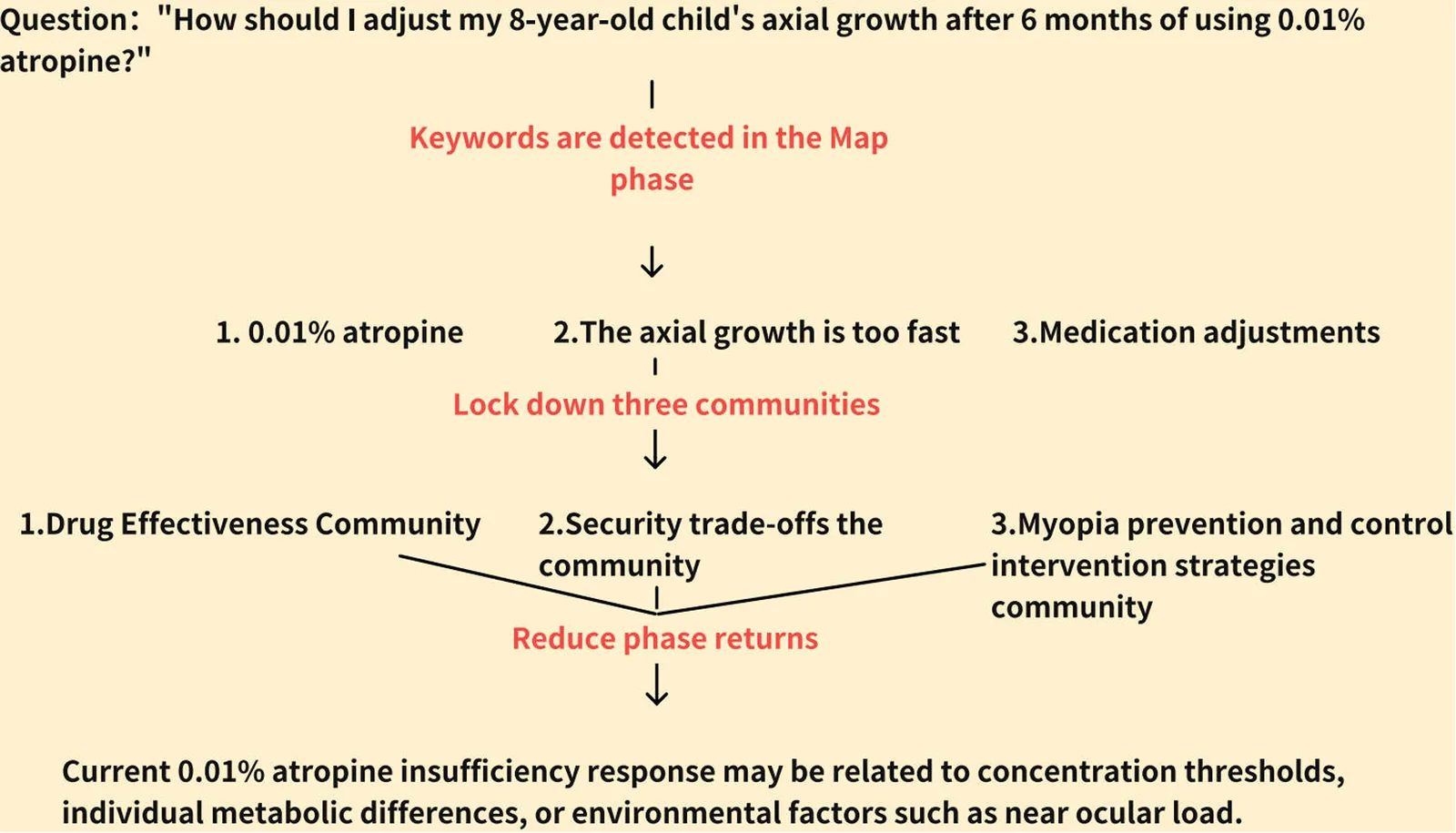
Example of question—answering implementation.

When a user poses a question, the system will conduct information retrieval by combining local and global searches. Based on the entities and relationships extracted from the knowledge graph, as well as the relevant text fragments in the original document, the system generates answers that are relevant to the specific question. For questions that require understanding of the entire dataset or broader topics, the system employs global search and aggregates multiple community reports using the MapReduce method, ultimately generating the answer (Figure 3). More specific parameter values or key words are provided in the Supplementary Materials.

## 3 Evaluation method

In order to comprehensively evaluate the performance of the MyoATP-AI model, we employed multiple evaluation methods. While using the results generated by artificial intelligence, we also combined manual assessment, aiming to obtain the model's accuracy, relevance, and the authenticity of the actual application data of the generated answers.

### 3.1 Evaluation of BLEU and ROUGE scores

In automatic evaluation, BLEU and ROUGE are used to measure the similarity between the generated text and the reference answers. During the automatic evaluation process, we use BLEU and ROUGE to measure the similarity between the generated text and the reference answers. The BLEU score mainly depends on the degree of n-gram matching, and the closer it is to 1, the higher the translation quality (28). The BLEU was caculated using Equation 1.

$$\mathrm{BLEU}=\mathrm{BP}*\exp\;\left(\frac{1}{n}\overset{N}{\underset{i=1}{\sum }}\;P_{n}\right)$$ (1)

ROUGE (Recall-Oriented Understudy for Gisting Evaluation) (29) evaluates overlap at the word and subsequence levels, with ROUGE-1 and ROUGE-L as key metrics. The ROUGE-N was caculated using Equation 2.

$$\mathrm{ROUGE}\text{-}N=\frac{\sum S\in \{\mathrm{Reference}\;\mathrm{Summaries}\}\sum \mathrm{gra}m_{n}\in S\mathrm{Coun}t_{\mathrm{match}}(\mathrm{gra}m_{n})}{\sum S\in \{\mathrm{Reference}\;\mathrm{Summaries}\}\sum \mathrm{gra}m_{n}\in S\mathrm{Count}(\mathrm{gra}m_{n})}\;$$ (2)

Together, they provide a comprehensive evaluation of the model's performance in generating high-quality, structurally sound answers.

### 3.2 RAGAS evaluation framework

We employed the RAGAS (Retrieval-Augmented Generation Assessment System) evaluation framework (30). It focuses on model faithfulness, answer relevance, and contextual recall. The following are the primary RAGAS evaluation metrics utilized in this study:

Faithfulness: This metric measures the factual consistency between the generated answer and the given context and is calculated using Equation 3.

$$\begin{array}{c} F=\frac{\left|V\right|}{\left|S\right|} \end{array}$$ (3)

Answer Relevancy: This metric evaluates the correlation between the generated answer and the user's query. The answer relevancy score is calculated using Equation 4.

$$\begin{array}{c} \mathrm{AR}=\frac{1}{n}\overset{n}{\underset{i=1}{\sum }}\;sim(q,q_{i}) \end{array}$$ (4)

Context Recall: This metric measures whether the system has fully utilized the retrieved contextual information when generating an answer as caculated using Equation 5.

$$\mathrm{context}\;\mathrm{recall}=\frac{\left|\mathrm{Ground}\;\mathrm{truth}\;\mathrm{sentences}\;\mathrm{that}\;\mathrm{can}\;\mathrm{be}\;\mathrm{attributed}\;\mathrm{to}\;\mathrm{context}\right|}{\left|\mathrm{Number}\;\mathrm{of}\;\mathrm{sentences}\;\mathrm{in}\;\mathrm{ground}\;\mathrm{truth}\right|}$$ (5)

The evaluation metrics adopted in this study effectively reflect the system's performance in addressing the complex issue of preventing and treating myopia with low-concentration atropine.

### 3.3 Manual evaluation

Five ophthalmologists participated in the manual assessment, and their background characteristics are summarized in Supplementary Table S1. All experts have professional qualifications in ophthalmology, with an average clinical experience of 14.6 years (range: 8–20 years), and all have direct clinical experience in treating children with low-concentration atropine myopia.

Assessment process. The experts independently scored the responses generated by the system, covering five dimensions: safety, professionalism, fluency, comprehensiveness, and overall satisfaction. Before the formal assessment, all experts completed 30 min of standardized training, during which they reviewed and discussed the scoring criteria and representative examples for each dimension. Subsequently, each expert independently reviewed all 20 pairs of QA pairs in one session, with each pair taking approximately 5 to 10 min. The response results of MyoATP-AI and Mistral 7B were randomly shuffled and encoded with anonymous identifiers.

### 3.4 Test set construction

The test questions are mainly formulated based on real clinical scenarios in this field. There are mainly two types of usage scenarios: consultation-type questions for patients (such as efficacy, side effects, rebound after discontinuation) and professional-type questions for doctors [such as the results of each concentration group in the LAMP study, and the effects of different concentrations on equivalent spherical power (SE) and axial length (AL)]. The test questions are drafted by humans, and the reference answers (ground-truth) are written based on 104 evidence-based literature included. The writing style requires priority to provide specific numerical values (mean change, standard deviation, percentage decrease, *P* value), which should be consistent with the output format constrained by the system prompt words.

## 4 Results

In this study, we employed the GraphRAG technology and combined automated metrics with manual evaluations to conduct a comprehensive and systematic performance assessment of the MyoATP-AI system. The results demonstrated that the MyoATP-AI system performed exceptionally well in terms of accuracy, relevance, reliability, and context utilization. Moreover, the manual evaluation further confirmed the potential value of the MyoATP-AI system in actual clinical applications.

### 4.1 Automated evaluation results

#### 4.1.1 The scoring results of BLEU and ROUGE

In Table 1, the score of the model BLEU is 0.9479, which is very close to 1. This indicates that the translation quality of the MyoATP-AI system is extremely good. In the field of preventing and controlling the progression of myopia with low-concentration atropine, BLEU performed exceptionally well, indicating that the generated answers by this model conform to medical expression norms and are highly consistent. We use ROUGE-1 and ROUGE-L to evaluate the model's responses. ROUGE is a common evaluation metric in the field of question answering. ROUGE can measure the degree of matching between the generated results and the standard results. In this study, the ROUGE-1 score was 0.969, indicating that the MyoATP-AI system performed exceptionally well in terms of word-level matching and provided a comprehensive answer. The ROUGE-L score is 0.9677, indicating that the responses generated by the MyoATP-AI system are highly consistent with the reference answers, and the core information is highly coherent.

**Table 1:** Scoring outcomes of LEU and ROUGE.

Indicator	Average value
BLEU score	0.9479
ROUGE-1 score	0.9690
ROUGE-L score	0.9677

#### 4.1.2 RAGAS evaluation results

We evaluated the performance of the retrieval-enhanced generation system using the RAGAS assessment framework. The RAG system enhances the accuracy of the MyoATP-AI system by integrating the retrieval module and the generation module. We quantitatively analyzed the performance of these two parts using the RAGAS framework and optimized the system accordingly. The specific experimental results are shown in Table 2.

**Table 2:** RAGAS evaluation results.

RAGAS	Results
Faithfulness	0.947
Answer relevancy	0.9587
Context recall	0.925

The accuracy score is 0.947, which indicates that the MyoATP-AI system can accurately generate responses from the retrieved literature. The answer relevance score is as high as 0.9587, indicating that the responses generated by the MyoATP-AI system are highly semantically relevant to the user's query and answer their current questions. The context recall score is 0.925, suggesting that the MyoATP-AI system can fully utilize the retrieved context information when generating responses, ensuring that the answers cover the key points related to the core of the question.

We speculate that these higher scores for lexical overlap are partly due to the narrow scope of the assessment and the restrictions on terminology. All questions and answers revolve around a set of common core terms (such as: 0.01%, 0.025%, 0.05% atropine, SE, AL, LAMP studies), and the output format of the system is structurally consistent with the reference literature written by experts, among other factors.

#### 4.1.3 Manual evaluation results

**Figure 4:**
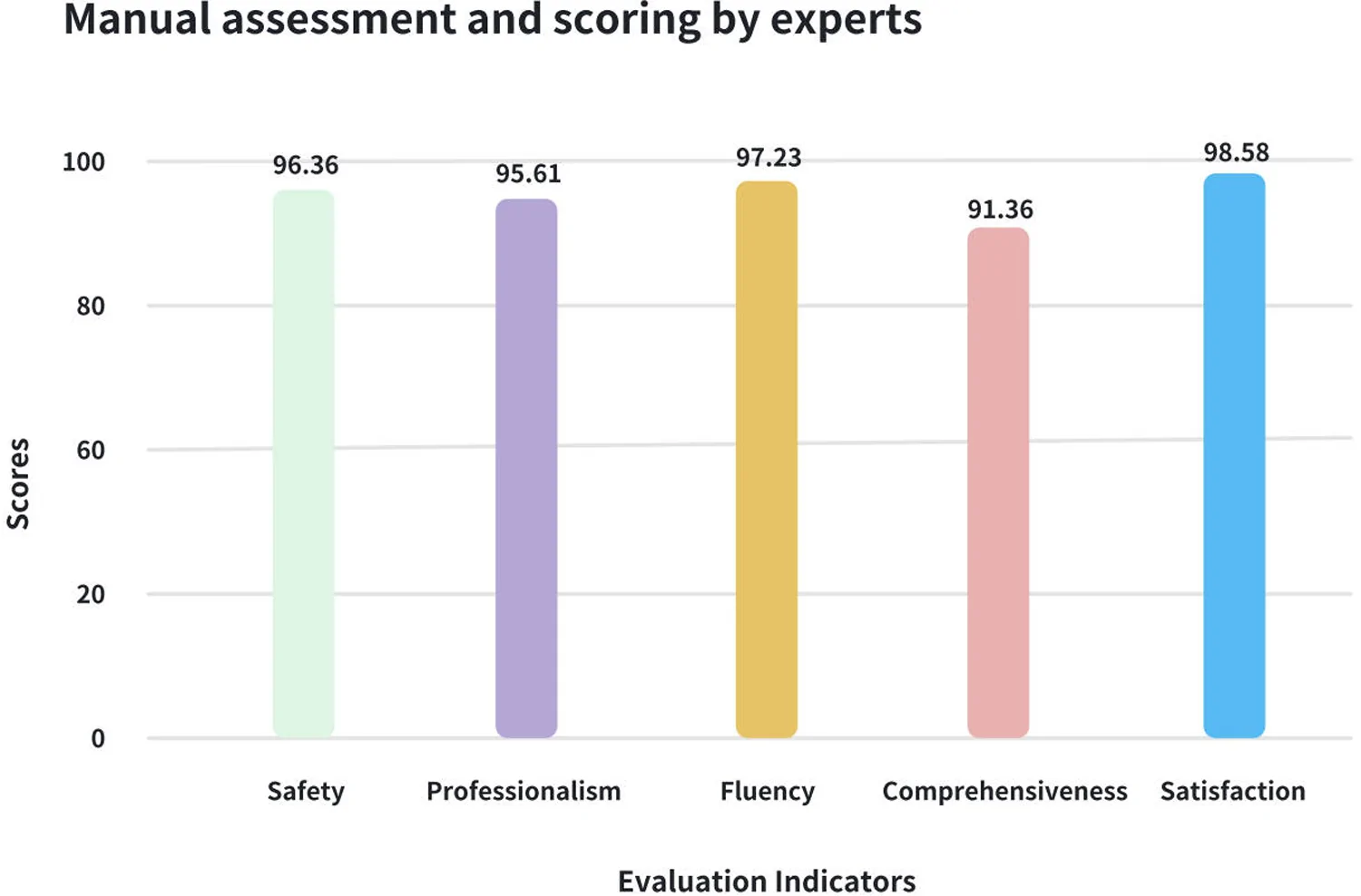
Manual evaluation results.

In addition to the automatic assessment, we also involved 5 ophthalmology experts for manual evaluation and scoring. The results showed that MyoATP-AI performed well in five aspects (Figure 4): safety, professionalism, fluency, comprehensiveness, and user satisfaction. The specific scores were: safety 96.36 points, professionalism 95.61 points, fluency 97.23 points, comprehensiveness 91.36 points, and user satisfaction 98.58 points. These results indicate that the MyoATP-AI system is highly reliable and practical in clinical practice and is recognized by ophthalmology experts. The high fluency score reflects that the language expression of MyoATP-AI is natural and easy for users to understand. The extremely high user satisfaction indicates that it is highly recognized in aspects such as clinical decision support, academic research, and patient education.

In conclusion, the results of the expert manual assessment fully demonstrate that MyoATP-AI excels in aspects such as safety, professionalism, smoothness, comprehensiveness, and user satisfaction. The MyoATP-AI system will provide strong support for the prevention and treatment of low-concentration atropine-induced myopia.

### 4.2 MyoATP AI: A comparative study against the 2024 consensus and general language models for myopia management

#### 4.2.1 MyoATP AI as a complementary evidence-update tool relative to the 2024 consensus framework

2024 expert consensus on the use of low-concentration atropine for myopia control represents the collective wisdom of top clinical experts. It is based on rigorous discussions, extensive clinical experience, and systematic evidence synthesis. This consensus remains the cornerstone of clinical practice and provides important and authoritative reference guidelines for frontline clinicians.

MyoATP-AI is not intended to replace or challenge this consensus-based framework. Instead, we position it as an auxiliary decision support tool with two specific functions: (i) evidence update—by continuously monitoring and integrating the latest literature, bridging the temporal gap between the consensus release and recent research; (ii) personalized extension—converting the general recommendations in the consensus into individualized, specific, and traceable reasoning paths, especially for complex or edge cases where the consensus may not provide clear guidance.

However, the final clinical decision must always be made by the attending physician, with the consensus serving as the main reference, and the AI-generated suggestions serving as Supplementary Information. We envision that future clinical workflows will benefit from the symbiotic relationship between the artificial intelligence system and the expert consensus, complementing each other and jointly leveraging their advantages (Table 3).

**Table 3:** Comparative analysis of MyoATP AI and the 2024 consensus.

Source	Evidence timeliness	Recommended concentration	Precision drug withdrawal
2024 consensus	With the literature cutoff date (2024-08-02) serving as the evidence lock point, its recommendations cannot incorporate new evidence emerging thereafter.	While 0.01% atropine sulfate is established as the benchmark concentration, the guidelines suggest considering concentration escalation for poor responders to 0.01%, yet fail to provide specific quantitative rules regarding concentration gradients, switching thresholds, or combination therapy protocols.	The probability of refractive regression after discontinuation of individual drugs has not been quantified. Consequently, clinicians cannot answer critical questions such as “when to discontinue treatment and what is the likelihood of a 0.5D/year progression post-cessation,” forcing them to rely solely on empirical judgment for trial discontinuation.
MyoATP AI	The daily-updated PubMed monitoring strategy (with a search range spanning from January 1, 2000, to May 31, 2025, incorporating 104 RCTs/prospective cohort studies) has compressed the half-life of the evidence base from “years” to “days,” significantly alleviating the issue of delayed integration of evidence-based medicine findings.	Twelve variables, including patient age, baseline spherical equivalent refraction, axial length growth rate, and photophobia score, were structured into nodal points. Using a Chain-of-Thought (CoT) reasoning engine, a decision pathway for “individualized concentration-frequency-combination strategies” was derived, enabling refined concentration recommendations that transition from “population stratification” to “monocular-level” precision.	By integrating global long-term follow-up data, an evidence-based optimal decision (continuation/dose reduction/discontinuation) can be determined, providing clinical guidance for precision-driven treatment cessation.

#### 4.2.2 accuracy comparison: MyoATP AI vs. general models

To compare the performance of MyoATP-AI and Mistral 7B in answering real-world clinical questions, we purposively selected four representative cases from actual outpatient encounters. These cases are divided into two categories: patient/parent-oriented queries (Table 4) and clinician-oriented queries (Table 5). The focus was on analyzing the efficacy, side effects, and safety of low-concentration atropine eye drops for childhood myopia. The comparison results highlighted the advantages of MyoATP AI in providing professional medical information.

**Table 4:** Example of a patient-oriented Q&A.

Question	MyoATP-AI'answer	Mistral 7B' answer
If my child uses these 0.05% atropine eye drops daily for two years, to what extent can this medication help slow down myopia progression compared to not using them?	In Chinese schoolchildren, daily use of 0.05% atropine eye drops for two consecutive years significantly controlled the progression of myopia. The results of the study showed that the mean change in equivalent spherical lens power (a measure of change in myopia) after two years was −0.46 ± 0.30 D in the atropine group, compared with −1.72 ± 1.12 D in the no placebo group. This means that myopia deepening was significantly slower in the atropine group. At the same time, the increase in axial length (the length of the anterior and posterior diameters of the eye, which is closely related to myopia) was also significantly less in the atropine group (0.26 ± 0.30 mm) than in the placebo group (0.76 ± 0.62 mm). Therefore, it was concluded that the use of 0.05% atropine for 2 years could effectively prevent elongation of the ocular axis and, thus, control the progression of myopia.	Slowing of myopic growth: Studies have shown that the use of 0.05% atropine eye drops in myopic children reduces the average annual progression of myopic refraction by about 0.18D to 0.39D. For example, myopia that might have increased by about 100 diopters per year may increase by about 50 to 70 diopters after use of atropine eye drops. Delayed axial growth: Long-term use of low concentrations of atropine can slow down the annual growth of axial length by about 0.06 mm to 0.25 mm. Generally, a 1D increase in myopia corresponds to an axial growth of about 0.3 mm, and thus slowing axial eye growth is very important in controlling the progression of myopia. In terms of myopia progression, 0.05% atropine was superior to 0.01% atropine. For example, the incidence of myopia and the percentage of patients with rapid myopic transition were significantly lower in the 0.05% atropine group than in the 0.01% atropine group.
What are the possible side effects of using 0.05% Atropine Eye Drops? Will myopia rebound quickly after stopping the medication?	The main side effect is mild photophobia (sensitivity to light), a discomfort that usually disappears within 7 days. Current studies have not reported severe allergies, headaches or rainbow rings in vision. Wearing photochromic lenses can help reduce photophobia. With regard to discontinuation, the study was conducted one year after discontinuing 0.05% atropine and found no significant rebound in axial length gain compared to the placebo group. The change in equivalent spherical lens power was also significantly lower in the atropine group than in the placebo group one year after discontinuation, suggesting that there may be a continuing inhibitory effect on myopia progression after discontinuation of the drug. In conclusion, the treatment with 0.05% atropine was well tolerated and had no significant adverse effects on quality of life.	There may be some side effects associated with the use of Atropine 0.05% Eye Drops, such as photophobia, blurred near vision or mild eye irritation in children. However, these side effects are generally not serious and are well tolerated by most children. Whether or not the myopia will return after stopping the medication varies from person to person. Some children may experience some rebound, but in general, the rebound with 0.05% atropine is relatively good.

**Table 5:** Example of a doctor-oriented Q&A.

Question	MyoATP-AI'answer	Mistral 7B' answer
What do the results of the first year of the LAMP study show about the effects of different low concentrations of atropine eye drops on myopia progression in children? Which of the three concentrations, 0.05%, 0.025%, and 0.01%, had the best effect at one year compared with placebo in slowing the progression of spherical equivalent (SE) and lengthening of the ocular axis (AL)?	Results from the first year of the LAMP study showed that 0.05%, 0.025%, and 0.01% atropine eye drops slowed myopia progression with a concentration-dependent response. After one year of treatment, the 0.05%, 0.025%, and 0.01% atropine groups showed 67%, 43%, and 27% reductions, respectively, in slowing the progression of mean spherical equivalent (SE) compared with the placebo group. In slowing the progression of ocular axis (AL) lengthening, these three concentrations reduced it by 51%, 29%, and 12%, respectively. Among the three concentrations, 0.05% atropine was the most effective in controlling SE progression and AL lengthening. Compared with the placebo group, the mean SE change in the 0.05% group was −0.27 ± 0.61 D compared with −0.81 ± 0.53 D in the placebo group (*P* < 0.001). The mean AL increase in the 0.05% group was 0.20 ± 0.25 mm compared with 0.41 ± 0.22 mm in the placebo group (*P* < 0.001).	Results from the first year of the LAMP study showed the following effects of different low concentrations of atropine eye drops on myopia progression in children: Slowed progression of spherical lens equivalent (SE): 0.05% concentration had the best effect at one year, slowing SE progression by 0.58 D compared to placebo. Slowed axial lengthening (AL): The 0.05% concentration was most effective at one year, slowing AL lengthening by 0.12 mm compared to placebo. In summary, the 0.05% concentration was most effective at one year in slowing SE progression and AL prolongation, AIh of which were significantly better than placebo.
The safety of low-concentration atropine eye drops and their side effects are of great concern to physicians and parents in pediatric patients. How well were the 0.05%, 0.025%, and 0.01% atropine eye drops used in the LAMP study tolerated? What are the common side effects and how do they compare to the placebo group?	The LAMP study showed that all three concentrations of low-concentration atropine eye drops (0.05%, 0.025% and 0.01%) were well tolerated in children. The decrease in accommodation was found to be minimal in all groups and had no significant effect on near and best-corrected distance visual acuity. Although there was a statistically significant difference in pupil dilation, the effect was minimal. The most common side effect was photophobia, which differed between groups at the 2-week visit (*P* < 0.001) but decreased over time at one year (*P* = 0.27). The need for photochromic spectacles and progressive lenses did not differ significantly between groups. A total of 13 serious adverse events requiring hospitalization were reported in the study, but none of these events were related to atropine use. Visual-related quality of life did not differ between all groups.	In the LAMP study, 0.05%, 0.025%, and 0.01% low-concentration atropine eye drops were well tolerated by most children. Common side effects included photophobia, blurred near vision, and ocular irritation, but these side effects were usually mild and transient. These side effects were seen in a slightly higher proportion of children using the lower concentration of atropine compared to the placebo group, but the overall safety profile was good with no serious adverse effects.

In terms of quantifying the therapeutic effect, MyoATP AI provides specific data from research, including the average change values, standard deviations, percentage reductions, and statistical differences in spherical equivalent refractive power and axial length. It directly assesses based on time or concentration requirements. In contrast, the data of Mistral 7B is vague and may contain inaccuracies.

Regarding side effects and safety, MyoATP AI provides detailed and professional answers. It lists common side effects, explains their duration, the impact on vision function and quality of life, management suggestions, and research data on rebound effects. It also includes detailed information on serious adverse events. However, the description of Mistral 7B lacks these in-depth contents and is rather superficial.

In conclusion, MyoATP AI outperforms Mistral 7B significantly when it comes to addressing professional medical issues such as pediatric myopia prevention. Its data-based, precise, detailed and informative responses can meet the diverse needs of users for reliable and in-depth professional medical information.

## 5 Discussion

Myopia progression in children/adolescents poses a global public health challenge. The exponential growth of low-concentration atropine research (spanning basic mechanisms to clinical studies) creates dynamically updated inefficient evidence to manually synthesize, often leading to missed critical data. Clinicians confronting complex or contradictory findings frequently resort to arbitrary or overly conservative concentration choices, failing to optimize therapeutic efficacy.

MyoATP AI is a novel evidence-based conversational AI system specifically designed for low-concentration atropine myopia management. Although GraphRAG and CoT have been applied in other medical fields, this study is one of the first to demonstrate the feasibility and clinical practicality of these methods in optimizing decisions related to atropine in children's myopia.

MyoATP-AI is an evidence-based decision support tool designed to provide structured, traceable, and evidence-based information and recommendations to clinicians, rather than a validated personalized treatment recommendation engine. Our evaluation directly confirmed that this system has the ability to retrieve and integrate evidence from published literature, generate responses consistent with expert opinions, and provide smooth and clinically relevant information. However, these capabilities do not equate to validated personalized treatment recommendations. Truly personalized treatment requires the integration of comprehensive patient-level clinical data, and more importantly, the combination of prospective intervention studies to demonstrate improvements in patient outcomes.

Based on this positioning, MyoATP-AI can contribute to clinical practice in multiple complementary ways. Firstly, it provides an up-to-date, transparent, and traceable atropine-related decision support tool for ophthalmologists, integrating the latest research findings and presenting information in a patient-centered manner. Secondly, the system provides reliable, accessible, and evidence-based medical information for patients and parents, helping them understand the progress of myopia, evaluate treatment options, and follow the recommended treatment plan. Thirdly, by shortening the evidence integration cycle from several years to nearly real-time, the system helps narrow the gap between research and clinical practice, ensuring that clinical decisions can refer to the most current scientific knowledge. Fourthly, although the system itself cannot guarantee cost reduction or alleviation of social burdens, it provides a path that enables more precise evidence-based decisions to potentially achieve these long-term goals, but such benefits still require prospective validation.

It is important to note that MyoATP-AI does not replace or challenge existing clinical guidelines. The 2024 consensus remains the cornerstone of clinical practice, providing an authoritative and rigorously validated framework. MyoATP-AI serves as a supplementary tool, updating evidence between consensus updates and extending general recommendations to specific patient situations. The final treatment decision—including concentration selection, adjustment, or discontinuation—is made by the attending physician, who must combine the system's recommendations with the patient's complete clinical status and individual preferences.

There are also many limitations in this study. First, lack of prospective clinical validation. All the recommendations generated by MyoATP-AI are based on the comprehensive analysis of retrospective evidence, rather than prospective intervention validation. The safety, effectiveness, and user experience of this system in real clinical environments have not yet been tested in practice. Our next steps is to deploy MyoATP-AI in clinical setting to test the practicality of MyoATP-AI. Second, limited comparison with other systems. Our comparative analysis is limited to the basic model (Mistral 7B) and does not systematically compare with other advanced medical large language models (such as GPT-4, Gemini, Claude) or existing AI tools related to myopia. Third, Size and construction of the test question set. The automatic evaluation is based on 20 knowledge question-answer pairs constructed by experts, and the sample size is relatively small. Additionally, as acknowledged earlier, some test questions overlapped thematically with the literature used for knowledge graph construction, which may have contributed to elevated lexical overlap scores (BLEU/ROUGE) and should be considered when interpreting these metrics. Fourth, limited expert validation of the knowledge graph. While multiple automated quality control mechanisms—including schema constraints, evidence grounding, entity normalization, and error logging—were implemented during knowledge graph construction, a formal, systematic expert review of the extracted triples has not yet been performed. Fifth, the expert safety assessment in this study (with a score of 96.36) provides valuable preliminary evidence for the safety of MyoATP-AI. However, we acknowledge that subjective scoring cannot fully replace objective safety verification. In future work, we plan to supplement expert assessment with a structured, quantitative safety assessment framework. We look forward to future research establishing a more rigorous and clinically meaningful safety verification framework for AI-based medical decision support systems.

**Figure 5:**
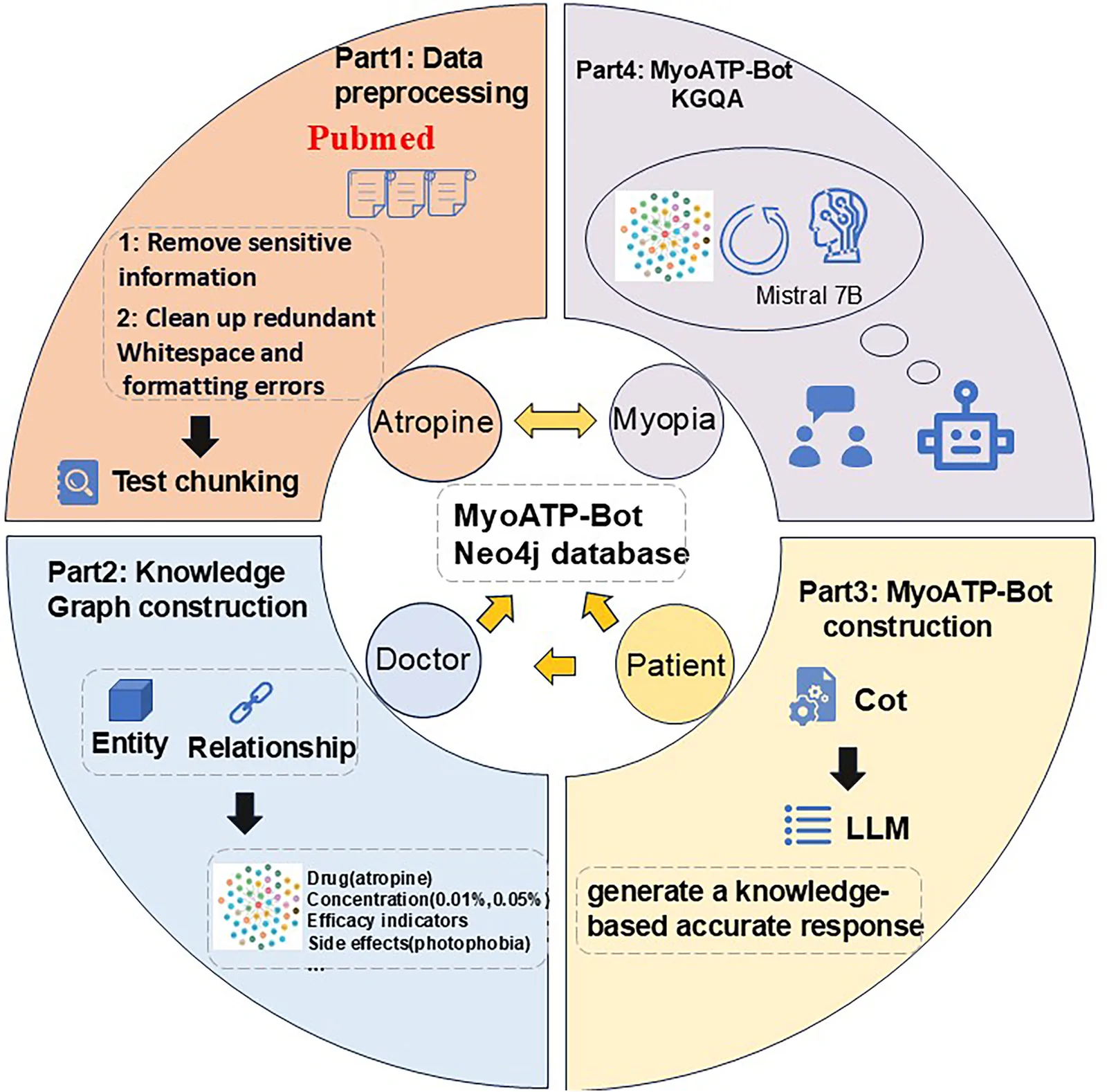
Flowchart of the construction process for the MyoATP-AI.

While atropine remains a cornerstone therapy, its personalized optimization represents a persistent research-clinic gap. Traditional evidence-based methods struggle in the era of information overload. Our study bridges this by innovatively combining large language models with GraphRAG and Chain-of-Thought (CoT) technologies, enabling precise, personalized, and dynamically optimized atropine therapy. The complete process is depicted in Figure 5.

## 6 Conclusion

This study successfully developed and validated an evidence-based conversational artificial intelligence system specifically designed for low-concentration atropine myopia control, demonstrating the feasibility and clinical value of applying GraphRAG and CoT reasoning techniques to a specific clinical field.

## Data Availability

Publicly available datasets were analyzed in this study. This data can be found here: https://github.com/hujili007/MyoATP-AI.

## Supplementary material

Supplementary Figure 1 Construction Process of Knowledge Graph: The 104 atropine articles were converted into TextUnits and segmented into 600-token blocks. The entities encompassed diseases, medications, diagnostic tools, therapeutic approaches, and so forth, while the relationships described the associations between these entities (for instance, the correlation between low-concentration atropine and myopia progression).

Supplementary Figure 2 Example of Knowledge Graph

Supplementary Figure 3 Flowchart for Community Analysis and Summarization of Knowledge Graph: These summaries not only provide a concise description of the entities and relationships within the community but also offer a hierarchical understanding of the system, facilitating subsequent question-answering generation. The reports encompass key entities and relationships within the community, and generate community summaries at various granularities through multi-level abstraction.

Supplementary Figure 4 Flowchart for Constructing Vector Representations of Documents
