# Supplementary material for: From static guidelines to dynamic personalization: a novel AI-driven evidence-based paradigm for low-concentration atropine prescribing in myopia control
Source: Front Med (Lausanne). 2026 Sep 7;13:1912484. doi: 10.3389/fmed.2026.1912484 (PMC13591467; doi:10.3389/fmed.2026.1912484)
Supplement: Supplementary file 1 [file Table1.docx]

All the numerical values are taken from the system configuration file (settings.yaml) and the process script, and can be accurately reproduced.

Text segmentation: The 104 pieces of literature are divided into text blocks of 600 tokens, with 50 tokens overlapping between blocks, resulting in a total of 1289 text blocks (TextUnits).

Large language model and generation parameters: Entity extraction, relationship extraction, community summary and question answering generation uniformly use Mistral-7B (deployed locally by Ollama), with a generation temperature of temperature = 0.2 to reduce randomness and ensure the stability and reproducibility of medical outputs.

Entity extraction: Limit to 10 types of entity types (Disease, Medication, Concentration, Symptom, AdverseEffect, DiagnosticTool, Treatment, Population, Study, Outcome). Types not included in this whitelist are all discarded in the model output. Finally, entities are extracted from 1289 text blocks, and after "merging entities with the same name and type, and merging aliases", 3581 unique nodes are obtained.

Relationship extraction: Limit to 18 types of relationship types (treats, associatedwith, reduces, increases, comparedwith, adverse_effect, dosage, population, causes, prevents, measures, indicates, improves, worsens, combination, duration, efficacy, safety). Each relationship is forced to carry an original evidence fragment (evidence span), and relationship types not in the whitelist are discarded. A total of 67,568 relationships are obtained.

Leiden community detection: Using the leidenalg library, the ModularityVertexPartition (modularity optimization) objective function is adopted, with the resolution parameter resolution = 1.0 (default value), and hierarchical division is performed at three levels (0, 1, 2) to obtain entities in different granularities and generate 7,149 community summaries based on this.

CoT prompts: The system integrates zero-shot CoT and few-shot CoT simultaneously. Zero-shot CoT adds "Let's think step by step" before the final answer to guide the model to perform internal reasoning first; few-shot CoT provides demonstrations to guide the model to analyze step by step according to the clinical path "identify the patient's condition → evaluate atropine solution (concentration/frequency) → check side effects → give comprehensive suggestions". It should be noted that the reasoning chain is not displayed in the final user answer, only the conclusion obtained through reasoning is retained to ensure concise and professional output (this point is also clearly stipulated in the question prompt template).
